# The Preoperative CT-Scan Can Help to Predict Postoperative Complications after Pancreatoduodenectomy

**DOI:** 10.1155/2015/824525

**Published:** 2015-10-29

**Authors:** Femke F. Schröder, Feike de Graaff, Donald E. Bouman, Marjolein Brusse-Keizer, Kees H. Slump, Joost M. Klaase

**Affiliations:** ^1^Department of Surgery, Medical Spectrum Twente, Haaksbergerstraat 55, 7513 ER Enschede, Netherlands; ^2^Faculty of Science and Technology, University of Twente, Netherlands; ^3^Department of Radiology, Medical Spectrum Twente (MST), Enschede, Netherlands; ^4^Twente Medical School, Medical Spectrum Twente (MST), Enschede, Netherlands

## Abstract

After pancreatoduodenectomy, complication rates are up to 40%. To predict the risk of developing postoperative pancreatic fistula or severe complications, various factors were evaluated. 110 consecutive patients undergoing pancreatoduodenectomy at our institute between January 2012 and September 2014 with complete CT scan were retrospectively identified. Pre-, per-, and postoperative patients and pathological information were gathered. The CT-scans were analysed for the diameter of the pancreatic duct, attenuation of the pancreas, and the visceral fat area. All data was statistically analysed for predicting POPF and severe complications by univariate and multivariate logistic regression analyses. The POPF rate was 18%. The VFA measured at umbilicus (OR 1.01; 95% CI = 1.00–1.02; *P* = 0.011) was an independent predictor for POPF. The severe complications rate was 33%. Independent predictors were BMI (OR 1.24; 95% CI = 1.10–1.42; *P* = 0.001), ASA class III (OR 17.10; 95% CI = 1.60–182.88; *P* = 0.019), and mean HU (OR 0.98; 95% CI = 0.96–1.00; *P* = 0.024). In conclusion, VFA measured at the umbilicus seems to be the best predictor for POPF. BMI, ASA III, and the mean HU of the pancreatic body are independent predictors for severe complications following PD.

## 1. Introduction

In Netherlands, each year more than 2000 patients are diagnosed with pancreatic cancer, mostly located in the pancreatic head [1]. The only curative treatment is a pancreatoduodenectomy (PD). Complication rates after pancreatic resections are up to 40%. Currently, BMI (>25 kg/m^2^) is considered as an easy to measure patient related factor associated with an increased risk of postoperative morbidity and mortality [2]. Since BMI does not necessarily reflect the distribution of fat, recent studies investigated the predicting probability of visceral fat area (VFA) and this measure seemed to be a more promising parameter to predict surgical outcome after pancreatic resection [2–5].

Another well-known factor associated with postoperative complications is a small pancreatic duct [6, 7]. Roberts et al. [7] developed a predictive score for complications following PD with an accuracy of 75% combining size of the pancreatic duct with BMI. Other investigations have shown that a fatty pancreas, also called pancreatic steatosis, is a risk factor for postoperative complications [3, 6]. Mathur et al. [6] examined the histology of the pancreas and found that a fatty pancreas was related to complications following PD.

The aim of the present study was to develop a predictive score for POPF and severe postoperative complications following PD. Therefore, the impact of different patient, tumour, and CT-derived data was analysed.

## 2. Methods

Patients undergoing PD or pylorus preserving PD (PPPD) at Medical Spectrum Twente between January 2012 and September 2014 were retrospectively identified from the hospital's medical database (*n* = 134). Patients from whom preoperative CT imaging was unavailable or incomplete were excluded from the study (*n* = 24). Preoperative data was gathered for each patient including gender, age, BMI, Charlson index, ASA score, and CT-data. Perioperative data included type of operation, duration of surgery, and perioperative blood loss. Postoperative data included localization of the tumour, histology of the tumour, radicality of resection, duration of the hospital stay, intensive care unit (ICU) admission, readmission, and complications up to 30 days after surgery. POPF was scored according to the classification system of the International Study Group of Pancreatic Fistula (ISGPF) [8]. The severity of complications was scored using the Clavien-Dindo classification of surgical complications [9, 10]. In this study, severe complications were defined as a Clavien-Dindo score grade IIIa or higher.

All used CT scans were postcontrast in the portovenous phase, with slice thickness ranging from 1 to 5 mm. The diameter of the pancreatic duct, attenuation of pancreatic tissue calculated as HU of the head, body, and tail of the pancreas, and the VFA were measured with a software program for CT, TeraRecon (Aquarius; TeraRecon, USA). This software enables semiautomatic measurements of a specific region with specified HU. The diameter of the pancreatic duct was measured perpendicular to the duct in the neck of the pancreas, obtained at the level of the confluence of the superior mesenteric and portal veins. The mean HU of the pancreas head, body, and tail was measured by manually drawing a region of interest (ROI) in these regions. The ROI was in the proximity, but did not include the pancreatic duct. The minimum size of the ROI was 1 cm^2^, but it preferably included an area as large as possible of homogeneous pancreatic tissue. VFA measurements were performed at three different levels, at the coeliac trunk, umbilicus, and top of the iliac crest.

*Statistical Analysis*. Data was analysed with IBM SPSS statistics version 22. Continuous data are presented with mean and standard deviation (STD) when normally distributed or median and range (IQR) when not normally distributed. Categorical data are summarized by frequency and percentage within each cohort. The univariate associations between variables and the different groups (no POPF versus POPF and nonsevere versus severe complications) were assessed using student's *t*-test or Mann-Whitney *U* tests for continuous variables. Categorical variables were compared by Pearson chi-square and Fischer's exact test. A *P* < 0.05 was considered statistically significant. Variables with a *P* < 0.15 in univariate analysis were entered in a forward stepwise multivariate logistic regression analysis to identify independent predictors for POPF or severe complications, based on the variables that were included in the multivariate model when they increased the fit of the model (based on the −2 log likelihood).

Per- and postoperative variables such as blood loss, length of stay, and readmission were not included in the multivariate logistic regression model, because these factors will not contribute to a preoperative risk prediction.

## 3. Results

The study cohort consists of 110 patients, 62% male, with a mean age of 66 years (±9.3). In the cohort, 47% of patients had a normal BMI (<25 kg/m^2^), 42% were overweight (25–30 kg/m^2^) and 11% were obese (>30 kg/m^2^). Of the patients, 36% underwent PD and 64% PPPD. The median operation time was 156 min (140–179 min) with an intraoperative blood loss of 500 mL (300–763 mL). The majority of patients had a pancreatic adenocarcinoma (47%) or periampullary carcinoma (35%). Other patients had neuroendocrine tumours (4%), or other (14%). The overall rate of POPF was 18%. Of all pancreatojejunostomy anastomotic leaks, 2 of them were graded POPF A, 9 of them POPF B, and 9 of them POPF C. By definition for POPF C reintervention was required; this group is within the severe complication group. No or nonsevere complications (Clavien-Dindo grades I-II) were seen in 67% of the patients. Severe complications, Clavien-Dindo grade ≥ IIIa, occurred in 33%. The postoperative mortality rate was 6.4%. Patient cohort characteristics are given in Table 1. There were no statistical differences in baseline characteristics between patients with and without POPF. However, for the selection of variables for the multivariate model to predict the occurrence of POPF (*P* < 0.15), BMI showed to be somewhat higher in patients with POPF (*P* = 0.13).

In univariate analysis, patients who experienced severe complications were more likely to have a higher BMI (*P* < 0.01). Patients who encountered severe complications had an increased median length of stay from 12 to 22 days (*P* < 0.01) and an increased risk of mortality from 0 to 19% (*P* < 0.01).

Preoperative CT measured values and resulting POPF and no/nonsevere or severe complications are given in Table 2.

Patients who developed POPF were more likely to have a higher VFA measured at the level of the coeliac trunk, umbilicus, and top of iliac crest (all *P* < 0.05). Patients who developed severe complications were more likely to have a lower mean HU of the pancreas head, body, and tail (all *P* < 0.05). Furthermore, patients with severe complications were more likely to have a higher VFA measured at the level of the coeliac trunk, umbilicus, and top of iliac crest (all *P* < 0.05). Pancreatic duct diameter was not associated with POPF or severe complications (*P* = 0.444 and *P* = 0.420).

BMI (OR 1.24; 95% CI = 1.09–1.42; *P* = 0.001), ASA class III (OR 17.10; 95% CI = 1.60–182.88; *P* = 0.019), and mean HU of the body of the pancreas (OR 0.98; 95% CI = 0.96–1.00; *P* = 0.024) were independent predictors for postoperative severe complications after multivariate analysis. With these variables, a risk score is developed as follows:

(1) $$\begin{array}{c} \frac{e^{\left(-4.801+0.215\left[BMI\right]+2.839\left[ASA\right]-0.02\left[HU\;\;body\right]\right)}}{1+e^{\left(-4.801+0.215\left[BMI\right]+2.839\left[ASA\right]-0.02\left[HU\;\;body\right]\right)}}. \end{array}$$

The risk score is based on the coefficient of the variables and the constant coefficient of the multivariate model (Table 3). To use this risk score the BMI, ASA score, and the mean HU of the body of the pancreas of the patient are needed. For correct use, ASA I and ASA II are filled in as 0 and ASA III as 1, and then a risk score between 0 and 1 is calculated. The risk score was validated with a 1000-sample bootstrap analysis (Table 4). These values were comparable to the values of BMI, ASA III, and HU in the multivariate model.

## 4. Discussion

This study reviewed various factors associated with the occurrence of POPF and severe complications after PD or PPPD. The main findings were that VFA measured at the umbilicus is the best predictor for POPF. BMI, ASA class III, and the mean HU of the pancreas body were independent predictors for postoperative severe complications.

The appearance of POPF is comparable to the rates found in previous studies [2, 5, 7]. However, POPF rates found in the literature range between 3.7% and 39%, which is probably caused by the various interpretations of POPF, despite an international consensus. However, as was stated by Gebauer et al. [11], there are some limitations in applying this fistula classification [8, 12]. Because of the differences in reporting of POPF, it is believed that the well-defined Clavien-Dindo classification is a more valuable tool to score postoperative complications as this classification tool is less subject to interpretation than the scoring of POPF.

The postoperative severe complication rate of 33% in this study was higher than the rates of 16.7–27.1% reported in literature [5, 13–15]. Multiple factors were significant after univariate analysis for nonsevere versus severe complications (Tables 1 and 2). After multivariate analysis (Table 3), BMI, ASA class III, and mean HU of the body of the pancreas remained predictors for developing severe complications after PD or PPPD. A risk score based on these three factors was made (1) and validated with a bootstrap analysis (Table 4). The advantage of these three factors as predictors is that these factors are preoperatively known or easy to measure. Currently, BMI and ASA are defined by the anaesthesiologist before surgery. Preoperative CT-scans are almost always available as part of preoperative staging. The HU of the pancreatic body can be easily measured by the surgeon or radiologist. Knowledge of a presumed high risk for POPF and/or severe complications could lead to change in intraoperative steps as performing pancreatogastric instead of pancreatojejunal anastomosis or using an isolated roux limb for the pancreatic anastomosis [16, 17]. Another possibility is a prehabilitation program to increase the anaerobic threshold, which might reduce the chance of complications [18, 19].

The BMI of the patients who developed severe complications was significantly higher. After multivariate analysis, BMI remained a valuable predictor for postoperative severe complications. This is in accordance with the expectations and literature [2, 5, 7, 20, 21]. In this study population, there were only 5 patients with an ASA class III classification, of whom 4 developed severe complications. However, to be able to say more about the quality of ASA class III as a predictor, more patients in the ASA class III category are needed. In other studies, investigating risk factors for the development of severe complications following PD or PPPD, ASA score is not frequently mentioned [2–5, 7]. Braga et al. [14] measured ASA score and analysed it for severe complications following PD. After multivariate analysis, ASA class III was found to be a significant predictor for developing postoperative severe complications.

This study showed that the mean HU of the body of the pancreas of patients who developed severe complications was significantly lower, compared to patients who did not. In a comparable study of McAuliffe et al. [5], nonenhanced CT-scans were measured and analysed to predict complications with Clavien-Dindo classes I–V. The mean HU of the pancreas for patients with complications scored Clavien-Dindo classes I–V was decreased but not significant (*P* = 0.130). This is probably due to the different classification of complications, McAuliffe et al. studied overall complications instead of severe complications. In the study of Roberts et al. [22], nonenhanced CT-scans were measured, the mean HU of the pancreas was found to be a significant predictor for POPF. As was the case in our present study, contrast enhanced images were used in the study of Hashimoto et al. [21]. They found the mean HU of the pancreas as a significant predictor for pancreatic anastomotic failure.

Next to the differences observed in the associations between complications and HU in the studies mentioned above, also the cause of lower HU can have various reasons. It is still unclear if fatty infiltration or steatosis, measured as a lower mean HU, is the cause of this phenomenon: washin and washout of contrast agent, namely, vary depending on the pathology of tissue and according to local blood flow mechanics [5]. Thus, the decreased HU could be attributed not only to steatosis or fatty infiltration of the pancreas but also to other reasons, such as a poorer blood flow and low cardiac output. Future studies should focus on noncontrast and postcontrast venous phase CT-scans and compare the measured HU of the pancreas.

Postoperatively, 18% of the patients developed POPF. After univariate and multivariate analyses, only the VFA remained as a significant predictor. In various studies, VFA was found to be a predictor for POPF [2–4, 20, 23]. However, in the majority of these studies, other factors were identified as predictors as well. Regularly, BMI and the pancreatic duct diameter were found to be predictors for POPF [7, 21, 22, 24]. A study by Roberts et al. [7] showed that a pancreatic duct diameter smaller than 3 mm increases the risk of POPF. In the present study population, the mean pancreatic duct diameter of the patient with POPF was smaller than the diameter of patients without POPF, although not significant. The literature is divided, since some studies indeed confirm a small pancreatic duct to be a significant predictor for POPF [6, 7, 24]. While others do not find a relation with POPF, even when the duct diameter is measured durante operationem [2, 4].

Despite the interesting and useful findings of our study, the present study also has several limitations. Firstly, the study included a variation of imaging protocols since patients were admitted from other different regional hospitals. The scans were obtained with different CT-scanners and the scan protocols varied depending on the patients referring hospital. Ideally, the measurement protocol uses a fixed scan delay with a specific contrast injection rate. Secondly, the effect of small timing differences between performance of scan and arrival of contrast in the structures was not taken into account. Thirdly, the used CT-scans had slice thickness ranging from 1 to 5 mm. For fat, muscle or HU measurement 5 mm slices were detailed enough. However, exact measurement of such small structures as the pancreatic duct may be diminished due to partial volume effects. Fourthly, from some patients, CT-scans were unavailable or incomplete; these patients were excluded. This could lead to a selection bias.

In conclusion, this study analysed preoperative CT images of patients who underwent PD or PPPD and investigated predictors for POPF and postoperative severe complications. VFA was found to be a significant predictor for POPF. The most significant factors to predict severe complications appear to be BMI, ASA, and the mean HU of the body of the pancreas. Based on these three variables, a risk score for postoperative severe complications after PD or PPPD was developed. To validate these results, a prospective study is required.

## Figures and Tables

**Table 1:** Patients cohort characteristics for POPF and nonsevere versus severe complications.

Factor	All	POPF		*P* value	Postoperative complications		*P* value
	(*n* = 110) (100%)	No (*n* = 90) (82%)	Yes (*n* = 20) (18%)		No or nonsevere (*n* = 74) (67%)	Severe (*n* = 36) (33%)	
Age, years, mean (STD)	66 (9.29)	66 (9.15)	65 (10.1)	0.733	65 (9.3)	68 (9.1)	**0.148**
Gender, *n* (%)				0.853			0.915
Male	68 (61.8)	56 (62.3)	12 (60)		46 (62.2)	22 (61.1)	
Female	42 (38.2)	34 (37.8)	8 (40)		28 (37.8)	14 (38.9)	
BMI (kg/m^2^), *n* (%)	25 (3.65)	24.8 (3.65)	26.2 (3.48)	**0.131**	24.4 (3.44)	26.8 (3.49)	**<**0.001^*∗*^
ASA, *n* (%)				0.402			**0.076**
I	5 (4.5)	4 (4.4)	1 (5)		4 (5.4)	1 (2.8)	
II	100 (90.9)	83 (92.2)	17 (85)		69 (93.2)	31 (86.1)	
III	5 (4.5)	3 (3.3)	2 (10)		1 (1.4)	4 (11.1)	
Charlson, *n* (%)				0.876			0.204
0	55 (50)	46 (51.1)	9 (45)		38 (51.4)	17 (47.2)	
1	31 (28.2)	25 (27.8)	6 (30)		23 (31.1)	8 (22.2)	
≥2	24 (21.8)	19 (21.1)	5 (25)		13 (17.6)	11 (30.6)	
Procedure, *n* (%)				0.639			0.169
PD	39 (35.5)	31 (34.4)	8 (40)		23 (31.1)	16 (44.4)	
PPPD	71 (64.5)	59 (65.6)	12 (60)		51 (68.9)	20 (55.6)	
Diagnosis, *n* (%)				0.196			0.972
Pancreatic adenocarcinoma	52 (47.3)	46 (51.1)	6 (30.0)		36 (48.6)	16 (44.4)	
Periampullary carcinoma	39 (35.5)	30 (33.3)	9 (45)		25 (33.8)	14 (38.9)	
Neuroendocrine tumour	4 (3.6)	4 (4.4)	0 (0)		3 (4.1)	1 (2.8)	
Benign diseases or tumour	11 (10.0)	7 (7.8)	4 (20)		7 (9.5)	4 (11.1)	
Other	4 (3.6)	3 (3.3)	1 (5)		3 (4.1)	1 (2.8)	
Radically, *n* (%)				0.24			0.641
R0	33 (30)	31 (34.4)	2 (10)		26 (35.2)	8 (22.2)	
R1	55 (50)	44 (48.9)	11 (55)		35 (47.3)	19 (52.8)	
R2	11 (10)	8 (8.9)	3 (15)		6 (8.1)	5 (13.9)	
n/a	11 (10)	7 (7.8)	4 (20)		7 (9.5)	4 (11.1)	
Surgical duration, min, median (IQR)	156 (140–179)	155 (140–179)	159 (145–174)	0.975	158 (142–179)	153 (138–177)	0.381
Blood loss, mL, median (IQR)	500 (300–763)	500 (300–700)	300 (550–975)	0.369	500 (300–663)	600 (400–975)	0.057
Length of stay, days, median (IQR)	12 (9–18)	11 (8–17)	17 (23–38)	<0.001^*∗*^	10.5 (8–14)	22 (14.5–34.5)	<0.001^*∗*^
Readmission, *n* (%)	27 (24.5)	23 (25.6)	4 (20)	0.602	14 (18.9)	13 (36.1)	0.049^*∗*^
Mortality, *n* (%)	7 (6.4)	5 (5.6)	2 (10)	0.609	0 (0)	7 (19.4)	<0.001^*∗*^

BMI: body mass index, ASA: American Society of Anesthesiologist, PD: pancreaticoduodenectomy, PPPD: pylorus preserving pancreaticoduodenectomy, STD: standard deviation, and IQR: interquartile range. ^*∗*^ *P* < 0.05 and bold are *P* < 0.015 and are included in the multivariate analysis.

**Table 2:** CT measured values for POPF and nonsevere versus severe complications.

Factor	All	POPF		*P* value	Postoperative complications		*P* value
	(*n* = 110) (100%)	No (*n* = 90) (82%)	Yes (*n* = 20) (18%)		No or nonsevere (*n* = 74) (67%)	Severe (*n* = 36) (33%)	
HU pancreas, HU, and median (IQR)							
Head	82 (69.4–98.6)	84.3 (70.2–98.4)	77.5 (63.4–98.9)	0.443	86.1 (73.5–99.7)	74.6 (58.1–93.5)	0.017^*∗*^
Body	79.4 (61.1–92.9)	79.4 (61.6–92.9)	76.8 (55.6–94.1)	0.541	80.5 (67.3–97.2)	74.5 (55–85)	0.014^*∗*^
Tail	80.4 (64.3–97.4)	79 (63.6–98.6)	81 (71.4–94)	0.947	83.2 (66.2–99.4)	74.1 (60–91.3)	**0.089**
Pancreatic duct, mm, median (IQR)	3.25 (0–5.06)	3.4 (0–5.1)	2.1 (0–4.8)	0.316	3.55 (0–5.1)	2.75 (0–4.79)	0.289
VFA, cm^2^, median (IQR)							
Truncus coeliacus	95.5 (59–142.5)	88 (50–135.8)	105.5 (85.6–178)	0.036^*∗*^	84 (48.3–129.8)	112 (75.5–155.3)	0.012^*∗*^
Umbilicus	130.5 (92.7–166)	119.5 (90–152.5)	157 (121.5–210.8)	0.008^*∗*^	118.5 (87.5–155)	147.5 (108.3–197.8)	0.016^*∗*^
Top of iliac crest	139 (96–204.3)	127.5 (93.8–184)	188.5 (118.8–242.8)	0.013^*∗*^	121 (89.8–182.3)	157 (116.3–232)	0.006^*∗*^

HU: Hounsfield units, IQR: interquartile range, and VFA: visceral fat area. ^*∗*^ *P* < 0.05 and bold are *P* < 0.015 and are included in the multivariate analysis.

**Table 3:** Multivariate analysis to predict severe complications.

Logistic regression for complications	Univariate			Multivariate			
	OR	95% CI	*P* value	OR	95% CI	*P* value	Coefficient
Age	1.034	(0.988–1.082)	0.149				
BMI	1.227	(1.084–1.390)	0.001	1.240	(1.086–1.415)	0.001	0.215
ASA I/II	ref (1.0)	—	—	—	—	—	
ASA III	9.000	(0.957–83.742)	0.054	17.095	(1.598–182.884)	0.019	2.839
Age	1.034	(0.988–1.082)	0.149				
HU pancreas							
Head	0.983	(0.967–0.999)	0.033	—	—	—	
Body	0.981	(0.966–0.997)	0.024	0.980	(0.962–0.997)	0.024	−0.020
Tail	0.985	(0.969–1.001)	0.062	—	—	—	
VFA							
Truncus coeliacus	1.007	(1.001–1.014)	0.030	—	—	—	
Umbilicus	1.009	(1.002–1.016)	0.016	—	—	—	
Top of iliac crest	1.007	(1.002–1.013)	0.011	—	—	—	
Constant						0.010	−4.801

BMI: body mass index, ASA: American Society of Anesthesiologist, VFA: visceral fat area, HU: Hounsfield units, OR: odds ratio, and CI: confidence interval.

**Table 4:** Bootstrap analysis to validate the risk score.

Bootstrap	OR	95% CI	*P* value	Coefficient
(1000 samples)				
BMI	1.240	(1.119–1.461)	0.001	0.215
ASA III	17.099	(1.629–162.00)	0.009	2.839
HU pancreas (body)	0.980	(0.954–0.998)	0.045	−0.020
Constant			0.004	−4.801

BMI: body mass index, ASA: American Society of Anesthesiologist, HU: Hounsfield units, OR: odds ratio, and CI: confidence interval.
